# Mast cell expression in oral submucous fibrosis: A systematic review and metanalysis

**DOI:** 10.4317/jced.60234

**Published:** 2023-07-01

**Authors:** Harshkant Gharote, Rahul Bhowate

**Affiliations:** 1 Professor, Oral Sciences Division, Dentistry Program, Batterjee Medical College, Jeddah, Saudi Arabia; 2 Professor, Department of Oral Medicine and Radiology, Sharad Pawar Dental College and Hospital, Sawangi, Wardha, 442004, Maharashtra, India

## Abstract

Background: Oral submucous fibrosis (OSMF) is a chronic disorder associated with reduced mouth opening, burning sensation and listed as potentially malignant disorder. The role of mast cells in initiation and progression of this condition has been debated in last few years. It is imperative to understand the definitive role of mast cells and subsequently identify a possible cost-efficient treatment modality for OSMF. This review aimed to study the role of mast cells in OSMF by framing a research question that assessed the mast cell count (MCC), their degranulation and immunohistochemical analyses. We performed a comprehensive search of PubMed, EBSCOhost and general Google search that conceded 26 studies from which 15 articles were finalized for the review. The individual study syntheses revealed increased MCC in OSMF as compared to controls. Also, there was decreased MCC with the progression of OSMF. However, the metanalysis showed high level of heterogeneity as three studies out of eight studies found reduced MCC in disease when compared with controls. There is definite increase in mast cell in OSMF although the cell count falls with the advancement of OSMF. This increases the scope for further research to identify exact mechanism by which mast cells contribute to fibrosis and conduct the drug trials that can inhibit the mechanism.

** Key words:**Oral submucous fibrosis, mast cell count, degranulated mast cells, stages of OSMF.Oral submucous fibrosis, mast cell count, degranulated mast cells, stages of OSMF.

## Introduction

Oral submucous fibrosis (OSMF) is a potentially malignant disorder (PMD) affecting oral mucosa and oropharynx and is frequently seen in Southeast Asia, especially the Indian subcontinent. This condition has an obscure etiology and cannot be cured completely, only symptomatic medical management can be rendered. Nonetheless, Areca nut is implicated as the main etiological factor, areca alkaloids strongly involve in the pathogenesis of OSMF (1,2). Over the last three decades, numerous studies had been performed to identify the molecular etiopathogenesis of OSMF including mast cell infiltration and mast-related biomolecules (3-5).

Sir Paul Ehrlich (1877) discovered a granular cell of loose connective tissue called mast cells; their functional behavior was almost completely linked to allergic conditions. Mast cells are bone-marrow-derived cells, and their mechanism is found to be complex, well-orchestrated, and multifunctional playing a significant role in the immune system (6,7). Mast cell progenitors exit the blood circulation to mature and inhabit the connective tissue, at the interface between the host and the outside environment, principally in subepithelial regions and in the connective tissue surrounding nerves, blood vessels, lymphatics, and mucus glands (7). Presently, it is well known that these cells play numerous roles in physiologic and pathological conditions including cancer angiogenesis (8).

Mast cells are strong contributors in the formation of classical proangiogenic factors like fibroblast growth factor 2, vascular epithelial growth factor, and non-classical factors like tryptase and chymase (9,10). The current information on mast cells, their count, and the various tumor-related angiogenic factors is quite largely available (8,11). Also, their count and role in certain PMDs like oral lichen planus have been studied extensively (12). However, the information about mast cells and their role in the pathogenesis of OSMF is sparse. With this viewpoint, the current systematic review was undertaken to delineate mast cell count (MCC) and their possible clinicopathologic implications in OSMF.

-Objectives

The primary objective of the present review was to assess the relationship between mast cell density/count and OSMF. Secondary objectives included if additional parameters like micro- vessel density, stage-wise MCC analysis in OSMF, and comparison with any other PMD or oral squamous cell carcinoma (OSCC) are estimated.

-PICOS

The keywords for the systematic review were defined as PICO format:

i) Population: “OSMF patients”

ii) Intervention: “mast cell density or count immunohistochemistry/serum analysis “

iii) Comparison: “healthy controls”, “normal healthy individuals”

iv) Outcomes: Assessment of MCC 

We constructed a primary research question using mentioned keywords: Is there any increase in MCC/density in OSMF? The secondary research questions included: a) “is there any degranulation of mast cells?’ and “is there any variation in the count with the progression of the disease”.

## Material and Methods

A systematic review of data published on mast cell count and the OSMF was performed following the preferred reporting items for systematic reviews and meta-analyses (PRISMA) guidelines.

-Literature searches and Eligibility criteria

The target population was OSMF patients who participated directly or indirectly (retrospective tissue analysis) for mast cell counting. PubMed, EBSCOhost, and Google search engines were searched using Boolean terms “mast cell”, and “Oral Submucous fibrosis” or “OSMF”. These terms were specified to “all fields” in the Advanced Search builder for PubMed and EBSCO. The search presented 14 articles from PubMed, 7 from EBSCOhost, and 9 from Google search until December 2021. While four articles were common in PubMed and EBSCOhost searches, those retrieved from Google search were not indexed in major indexing and abstracting systems.

-Data collection process

The decision to include the articles was made by reading the article title, abstract, and text containing the search terms and retrieved 26 articles for the review. The inclusion criteria were the presence of search terms, MCC performed between the study group and controls, and any additional data showing the relationship between mast cells and OSMF. The general review articles, prevalence studies, and other molecular studies on OSMF found during the search were excluded from the study.

-Data extraction

Both the authors independently extracted the data from each study and included items as the first author, year of publication, staining technique, key features, and inference of the study. Further, sample sizes, means, and standard deviations values of MCCs from each selected study were tabulated. In addition, data for the expression of mast cells in various stages of OSMF, other PMDs and OSCC were recorded. Any disagreement or data extraction errors were settled by reviewing the subtleties of that specific article by following the research question, inclusion and exclusion criteria, and study results.

-Quality assessment

Both the authors critically reviewed the selected articles for clarity of focused question, selection of study populations (no. of cases and controls), and study design. Further, validation of exclusion criteria, comparison of data to establish statistical differences, and applicability of result were assessed among these studies. The relevant information collected was based on the SIGN methodology checklist version 2.0 for case-control studies (Scottish Intercollegiate Guidelines Network) (13).

-Synthesis methods

The statistical analyses were performed using the Review Manager computer program (RevMan) version 5.4 for academic use recommended by Cochrane Collaboration, 2020 (14). The standard deviation for one study was calculated from the mean and *p-value* following the sixth chapter of Cochrane Handbook Guidelines (15).

## Results

-Study selection

Studies were selected for two data sets: key salient features exhibiting search terms and fulfilling the parameters of the primary research question. Data were also extracted if the studies further addressed the secondary research questions.

-Study characteristics

Study characteristics included the presence of a comparison of MCC between OSMF and normal tissue. Additional features like comparisons of MCCs with PMDs and OSCCs were also taken into consideration.

-Risk of bias in studies

Methodological assessment of studies selected as evidence was based on the SIGN methodology checklist (13). The checklist focused on specific aspects of the study design that research has shown to have a significant effect on the risk of bias in the results and conclusions. Eight studies were of high quality whereas seven studies were accepTable after the methodology checklist for case-control studies. Two papers published only data for the OSMF group and failed to address the primary research question. Thus, 15 studies were included in the review and eight high-quality studies were selected for metanalysis.

-Results of syntheses

**Figure 1:**
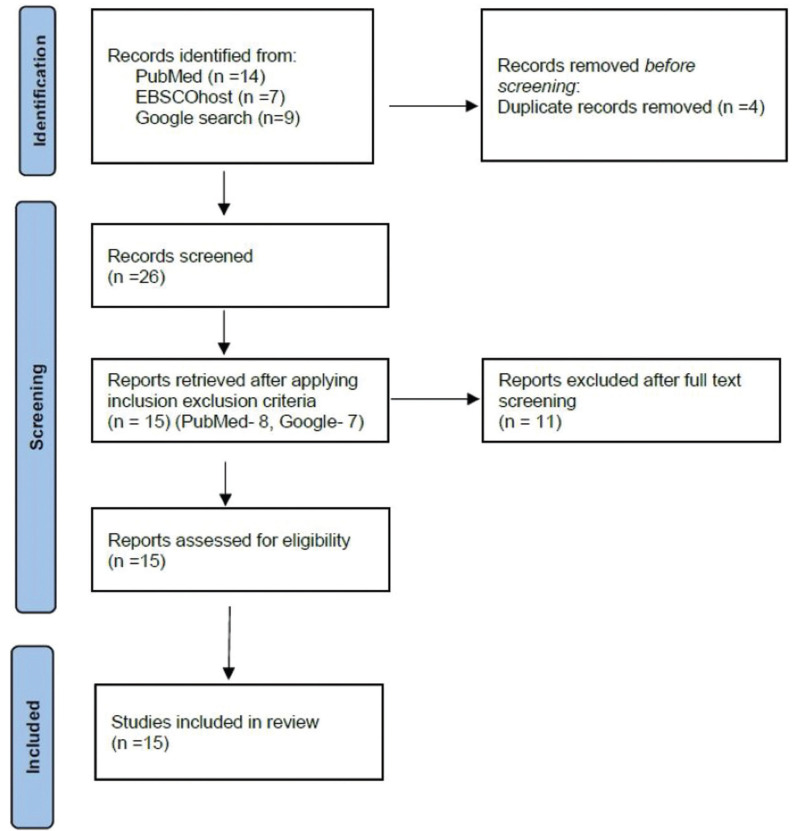
Flowchart illustrating the process of study selection for the systematic review.

A meticulous search yielded a total of 26 articles that were scrutinized according to inclusion and exclusion criteria. We excluded six articles from PubMed (4 general reviews), all three from EBSCOhost, and 2 from Google search as they did not match with the PICO format. Thus 15 articles were selected for final analysis as given in the flowchart (Fig. 1) as per PRISMA guidelines.

-Results of individual studies

A total of 12 studies used toluidine blue stain for MCC (4,16-26). Two studies used immunohistochemistry (27,28) while one article (29) used the toluidine blue staining and c-kit immunohistochemistry for comparison of mast cell expression (Table 1). Five studies (19,20,23,25,27) examined the degranulation of mast cells (Table 1) whereas seven articles (18-21,25,26,29) explored MCC in various stages of OSMF (Table 2). Six studies evaluated other PMD and/or oral squamous cell carcinomas for comparative evaluation of mast cells (4,17,22,24,27,28) (Table 3).

**Table 1:**
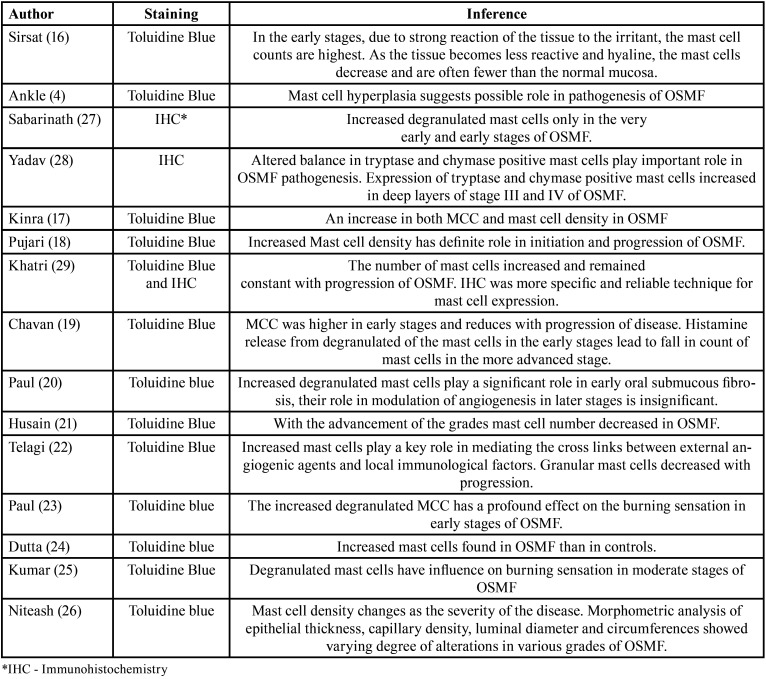
Summary of the articles selected for review.

**Table 2:**
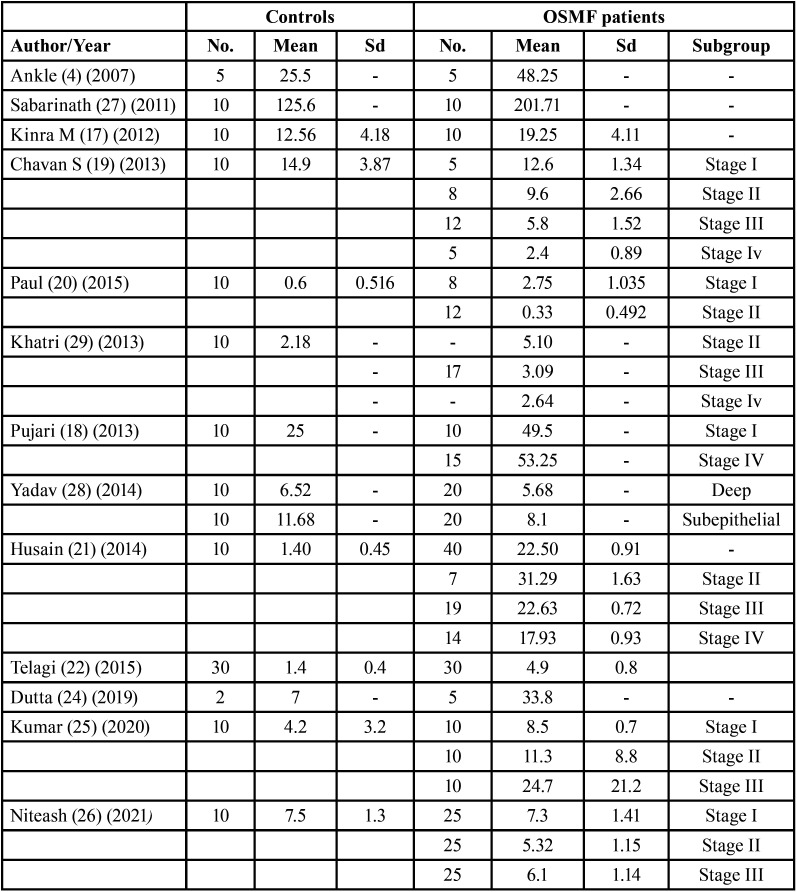
Characteristics of studies included in the review with means standard deviations and sample sizes in OSMF and controls.

**Table 3:**
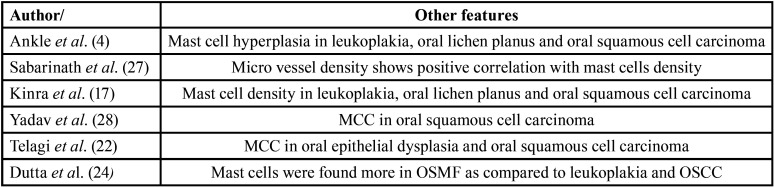
Summary of Studies that performed additional analysis with other potentially malignant disorders and oral squamous cell carcinoma.

**Figure 2:**
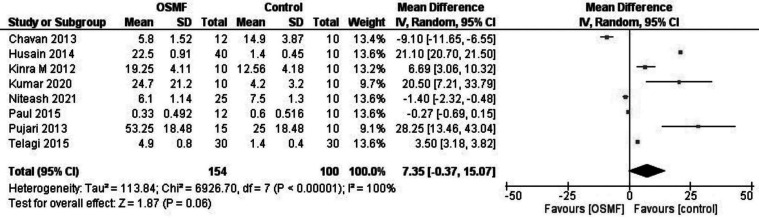
Forest plot of pooled effect size, estimates and 95% confidence interval representing differences in MCC between OSMF and controls.

Seven studies provided the sample size, mean and standard deviation for both groups, for one article standard deviation was calculated from the *p-value* (Table 2). Thus, a Meta-analysis based on data from 8 studies was performed using the RevMan for academics. The synthesis calculated the pooled average effect size, the standard deviation, and 95% confidence intervals. The forest plot revealed a significant variation in the MCC between OSMF and control groups (effect size = 7.35, *p-value* < 0.00001, z= 95% confidence interval: -0.37 to 15.07) (Fig. 2). However, the high I2 value of 100% indicates a high level of heterogeneity between the studies included.

## Discussion

OSMF is a chronic progressive disorder of the oral cavity, oropharynx, and esophagus which is induced primarily by betel nut through its alkaloids. The disease exhibits fibrosis that continues to cause difficulty in mouth opening, a burning sensation, and possesses high chance of malignant transformation. There are studies that have described etiopathogenesis through molecular pathways, trace elements, and mast cells (25,28,30). Although the first study (16) on MCC was performed in 1967, few studies have been performed in the last two decades to identify their role in OSMF.

The present systematic review observed a significant increase in MCC in OSMF. The forest plot of the meta-analysis suggested a significant increase as compared to controls. Sabarinath *et al*. proposed a possible role of inflammatory cytokines that stimulate the infiltration of the mast cells disease process (27). Ankle *et al*. stated a possible key role of mast cells in mediating the cross-talks between the external antigenic agent and the local immunologic factors (4). Telagi *et al*. attributed the increase in mast cells to vesicle formations and subsequent itching sensation due to histamine release (22). Further, Pujari *et al*. supported increased MCC to the association between mast cells and fibroblasts in the pathogenesis of OSMF (18).

A high level of heterogeneity in metanalysis was due to three studies (19,20,26) that showed negative inverse variances. These studies depicted a decrease in MCC in OSMF as compared to controls. Chavan *et al*. attributed the decreased count to an initial high inflammatory response of mast cells in the early stages as compared to fibrotic late stages which leads to the depletion of these cells in submucosa (19). Paul *et al*. suggested role shifts in mast cells causing a subsequent decrease in MCC (20).

Two studies observed an increase in MCC with the progression of the disease and predicted the possible role of mast cells in the initiation and progression of the disease however, no definite mechanism is proposed (18,25). Among five studies that found a decreasing trend in MCC, Chavan *et al*. attributed it to active degranulation of the mast cells causing continuous histamine release in the early stages leading to the scarcity of mast cells in the more advanced stage of the disease (19). Husain *et al*. and Niteash *et al*. correlated the decreased MCC to the inability of toluidine blue to stain degranulated mast cells and to decreased reactivity and hyalinization of the tissue respectively (21,26). On the other hand, Khatri *et al*. compared MCC in various stages of OSMF by toluidine blue and immunohistochemical staining and reported a higher count with c-kit staining, which was statistically significant in stage II and nonsignificant in stages III and IV of OSMF (29).

Although toluidine blue was the principal stain in most of the articles, three papers applied immunohistochemical analysis for MCC. Yadav *et al*. found that the tryptase-positive mast cells had a significant difference in stage I and other stages in the subepithelial zone while the controls exhibited a higher count in the deep zone compared to OSMF. Further, chymase-positive mast cells did not show statistically significant differences in the superficial zone while the deep zone revealed a significant difference in various stages of OSMF (28). Khatri *et al*. confirmed the high reliability of immunohistochemistry over the toluidine blue staining technique (29).

Kumar *et al*. assessed the relationship between the burning sensation in OSMF with degranulated mast cells and concluded that degranulated mast cells had a significant influence and were a positive predictor in moderate levels of burning sensation in OSMF (25). A study by Paul *et al*. also concluded a significant correlation between MCC and burning sensation (23). In another study, He observed a significant difference in the early stage while a nonsignificant difference in advanced stages in degranulated MCCs as compared to controls. Further, the correlation between intact and degranulated mast cells was highly significant (20). One study found a decrease in the degranulated MCCs as the progression from stage I to stage III of OSMF (27). Similarly, another study reported that continuous histamine release in the early stages causes a paucity of degranulated mast cells as the disease progresses (19).

Sabarinath *et al*. reported an increased microvascular density in OSMF than controls but found a nonsignificant positive correlation with MCC. Paul *et al*. found a significant difference in mean luminal diameter in the early and late stages as compared to controls (27). Similarly, Niteash *et al*. also demonstrated significant differences in the mean luminal diameter in various stages of OSMF (26).

Six studies included other PMD and/or oral squamous cell carcinomas for comparative evaluation of mast cells in OSMF (4,17,22,24,27,28). The PMD included leukoplakia and lichen planus. Except for Dutta *et al*. (24), all the studies reported increased count in PMD and OSCC compared to OSMF. One article performed MCC and lipid profiles in OSMF patients but there was no correlation mentioned in the study (21).

The present review disclosed that the MCC had a definite role in the pathogenesis of OSMF. However, the exact mechanism of these cells in initiation and progression was nonconclusive. Although certain studies tried to correlate the count with various stages and there was a significant fall in MCC with the severity of OSMF, the possible roles of cross-talks or entry of other biomarkers are obscure. OSMF possesses a high malignant transformation rate (30), therefore there is a need to identify proangiogenic biochemicals in the tissue microenvironment that stimulate mast cell proliferation in OSCC.

The systematic review puts forward various research opportunities for further biomolecular research in mast cell-related serine proteins (Chymase and tryptase), histamine, and diamine oxidase. The plasma levels of these molecules can give an insight to the systematic aspect of mast cells in OSMF. Mast cells in subepithelial tissues are considered gatekeepers, the chronic inflammatory response may change them to destructors. As Pujari *et al*. debated the therapeutic implications that can target the mast cell-endothelial cell axis to influence mast cell secretions to control inflammation and fibrotic changes. Drugs that can stabilize mast cells need to be considered for future treatment modalities (18).

## Conclusions

From this systematic review, it is evident that MCC is increased in OSMF that decreases with the progression of the disease. But the exact mechanism of action and biomolecular pathogenesis is obscure. There is a lack of data about the variation in MCC in pretreatment and post-treatment setting. Authors believe that there is a need for pre- and post-treatment studies targeting mast cells and correlating the outcomes with the MCC. Further, there is a need to understand the exact mechanism of action by mast cells in initiating malignant transformation in OSMF so that a target drug therapy can be instituted through clinical trials.
